# Supplementary material for: Cardiomyocyte Apoptosis Is Associated with Contractile Dysfunction in Stem Cell Model of MYH7 E848G Hypertrophic Cardiomyopathy
Source: Int J Mol Sci. 2023 Mar 3;24(5):4909. doi: 10.3390/ijms24054909 (PMC10003263; doi:10.3390/ijms24054909)
Supplement: Supplementary file 1 [file ijms-24-04909-s001.zip › ijms-2207704-supplementary.pdf]

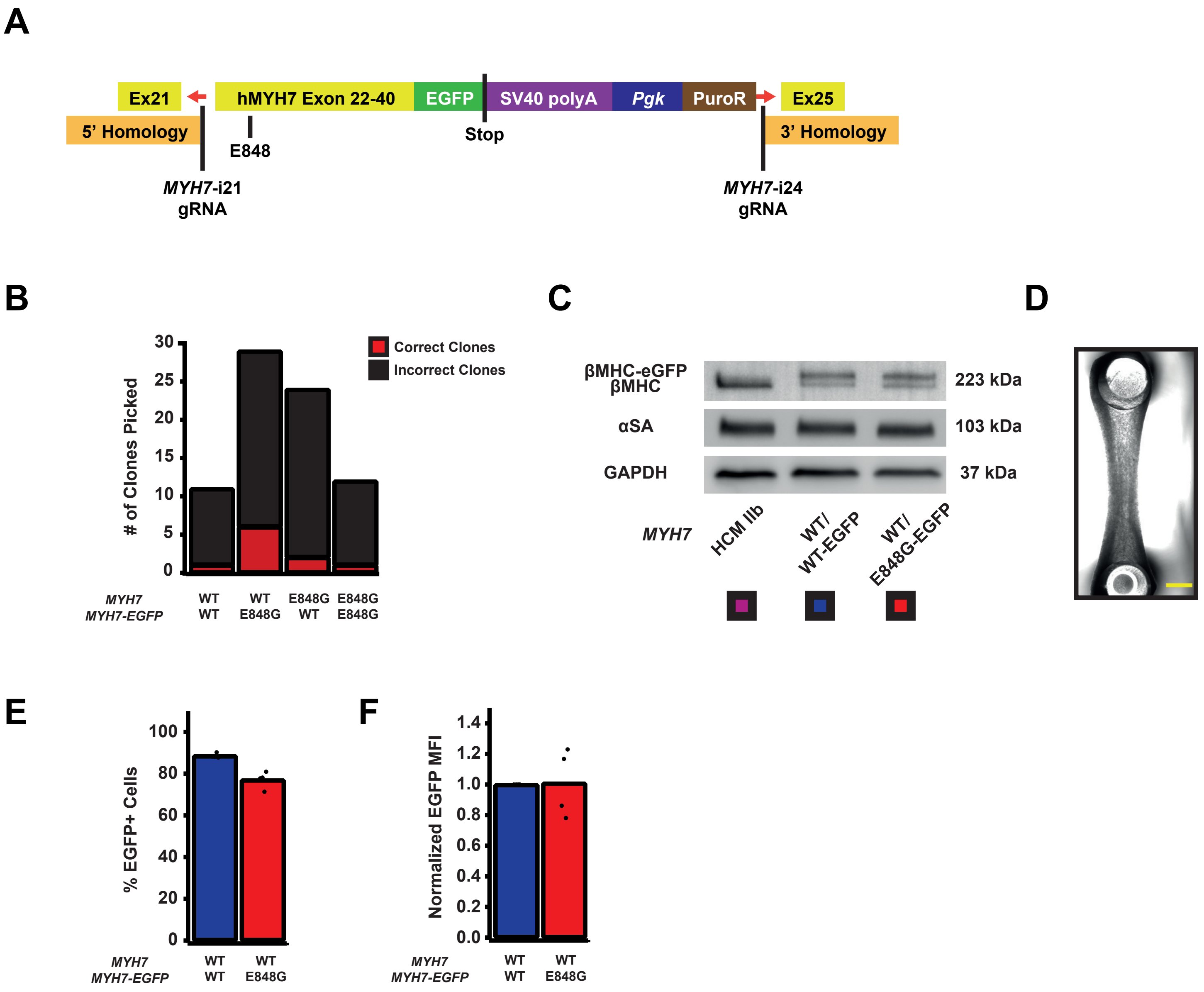


**Figure S1:** Generation of isogenic βMHC-EGFP expressing hiPSC-CMs using CRISPR/Cas9 editing (A) Schematic of pJet-MYH7-EGFP-PGK-PuroR plasmid with E848 location highlighted. (B) Number of clones picked with correct or incorrect edited sequence for isogenic lines. (C) Repre-sentative western blot for MYH7 protein (βMHC), α-sarcomeric actinin (αSA), and GAPDH protein expression in patient-derived and isogenic differentiation day 40 hiPSC-CMs, 20 µg total protein per lane. (D) Representative brightfield image of an engineered heart tissue attached to flexible (top) and stiff (bottom) posts. Scale bar 1 mm. (E) Total EGFP+ cells as percentage of total cells for dif-ferentiation day 25 hiPSC-CMs as measured by flow cytometry, n = 4 biological replicates. (F) EGFP mean fluorescent intensity in EGFP+ hiPSC-CMs for differentiation day 25 hiPSC-CMs as measured by flow cytometry, n = 4 biological replicates.

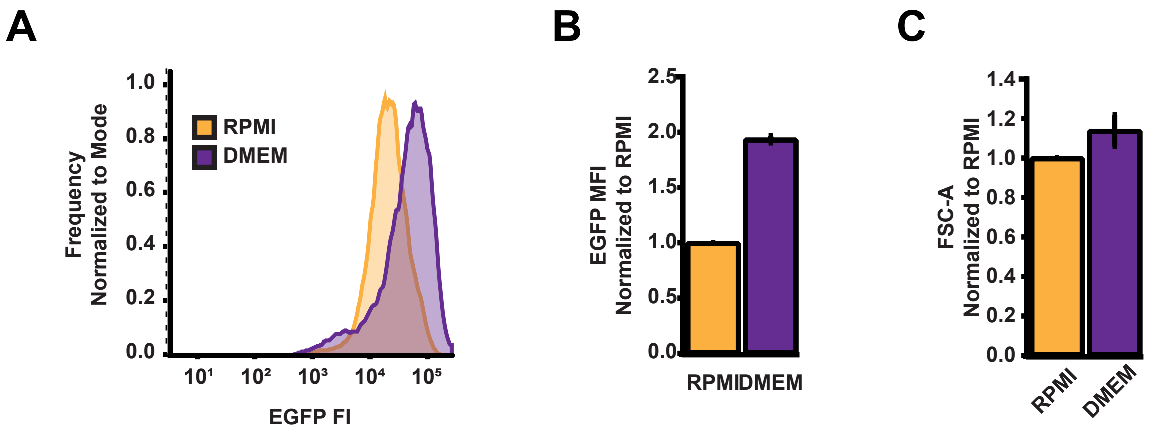


**Figure S2:** DMEM-based cardiomyocyte media accelerates maturation of hiPSC-CMs in monolayer culture
(A) Representative histogram of EGFP fluorescent intensity as measured by flow cytometry in MYH7WT/WT-EGFP hiPSC-CMs, differentiation day 40, after 10 days in RPMI-based and DMEM-based monolayer culture conditions. (B) Normalized EGFP geometric mean fluorescent intensity from (A). Mean ± SEM, n = 2 biological replicates. (C) Normalized FSC-A as measured by flow cytometry of EGFP+ MYH7WT/WT-EGFP hiPSC-CMs, differentiation day 40, after 10 days in RPMI-based and DMEM-based monolayer culture conditions. Mean ± SEM, n = 2 biological replicates. * in (B,C) indicates $p < 0.05$ significance calculated by Student's t-test.

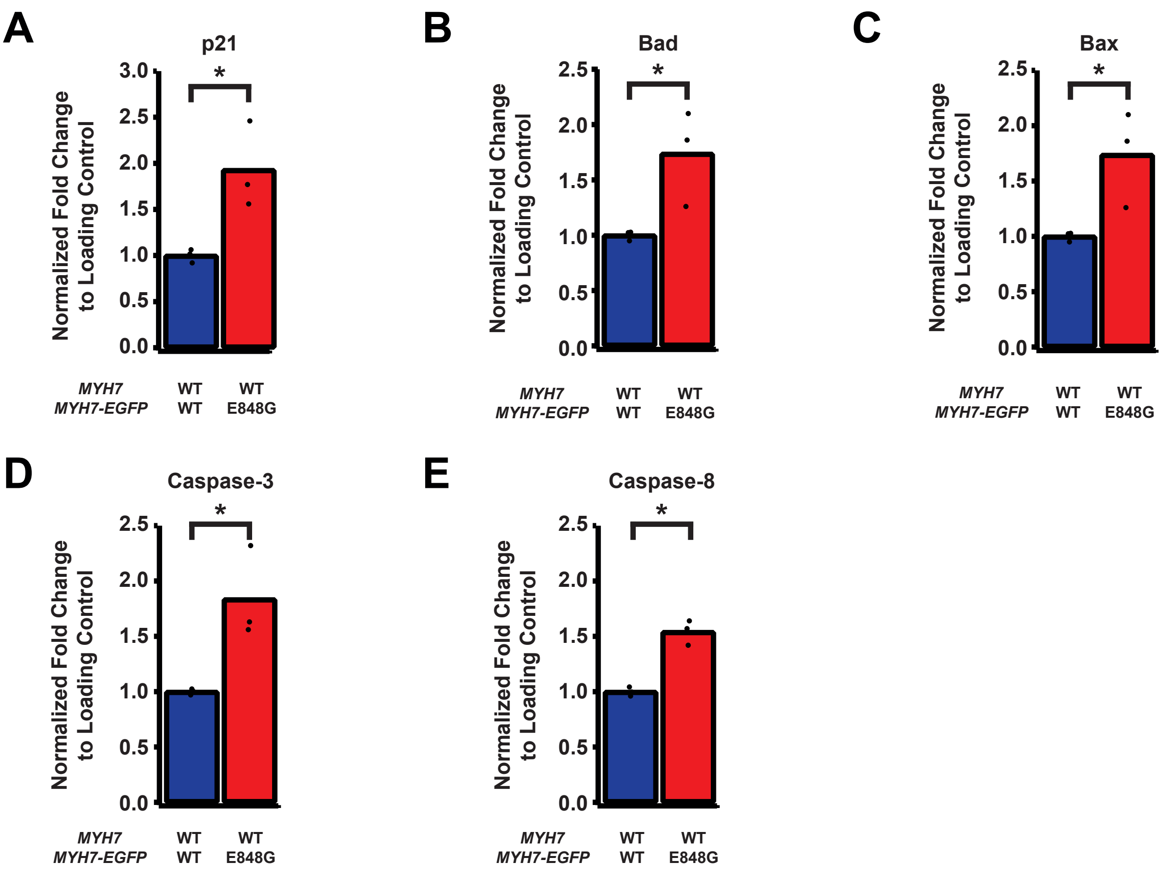


**Figure S3:** MYH7 E848G induces p53-associated intrinsic apoptosis in monolayer and EHT culture (A-E) Quantification of protein expression from Figure 3A normalized to loading control and MYH7WT/WT-EGFP. Mean and biological replicates shown, n = 3 technical replicates. * in (A-E) indicates $p < 0.05$ significance calculated by Student's t-test.

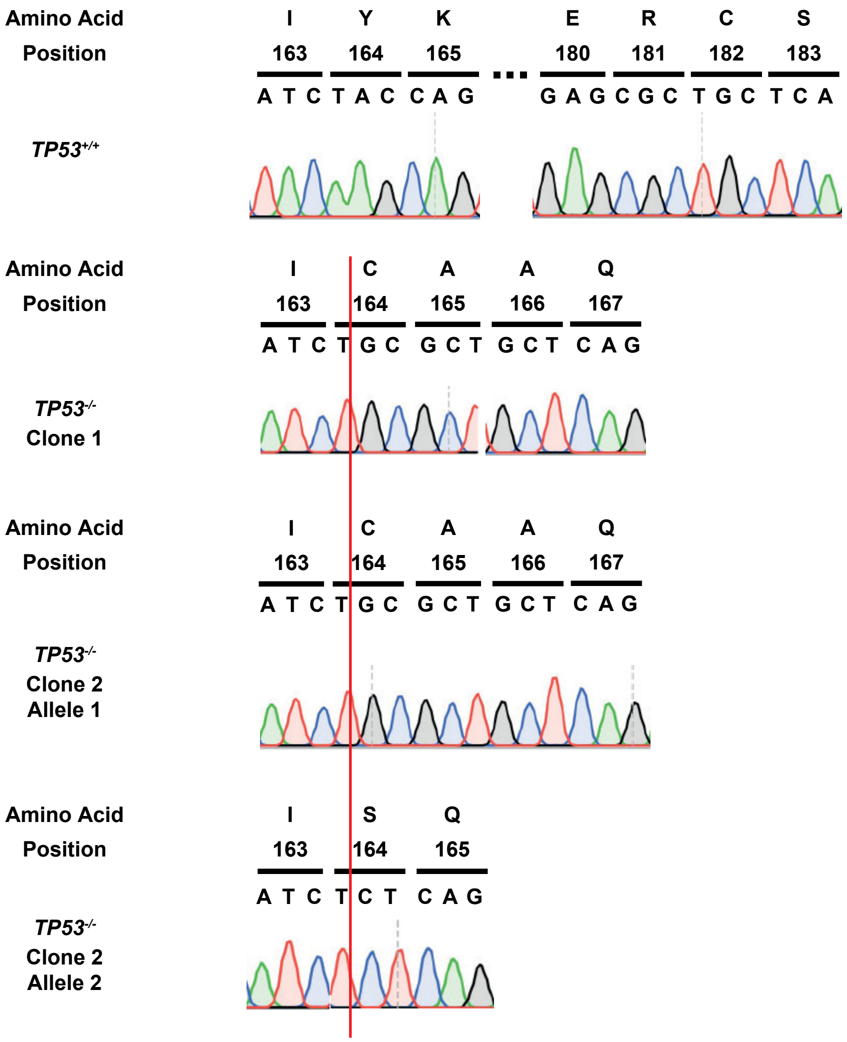


**Figure S4:** Sanger sequencing chromatograms of MYH7WT/E848G-EGFP TP53+/+ and MYH7WT/E848G-EGFP TP53-/- clones.
